# p21 can be a barrier to ferroptosis independent of p53

**DOI:** 10.18632/aging.103961

**Published:** 2020-09-24

**Authors:** Divya Venkatesh, Brent R. Stockwell, Carol Prives

**Affiliations:** 1 Department of Biological Sciences, Columbia University, New York, NY 10027, USA; 2 Department of Chemistry, Columbia University, New York, NY 10027, USA

**Keywords:** p21, ferroptosis, cancer, p53-independent, organ damage

## Abstract

Traditionally, the p21 protein has been viewed as limiting cancer progression and promoting aging. In contrast, there are reports that p21 can enhance cancer survival and limit tissue damage, depending on the tissue of origin and type of stressor involved. Here, we provide evidence to support these latter two roles of p21 by exploring its ability to regulate ferroptosis. Ferroptosis is a form of cell death that is associated with certain degenerative diseases, some of which are aging-related. Our results reveal a correlation between p21 protein levels in cell lines that are resistant to ferroptosis (p21 high) versus cell lines that are sensitive and easily undergo ferroptosis (p21 low). We also show that p21 levels themselves are differentially regulated in response to ferroptosis in a p53-independent manner. Further, experimentally altering the abundance of p21 protein inverts the ferroptosis-sensitivity of both resistant and sensitive human cancer cell lines. Our data also indicate that the interaction of p21 with CDKs is crucial for its ability to restrict the progression of ferroptosis. While this study was performed in cancer cell lines, our results support the potential of p21 to aid in maintenance of healthy tissues by blocking the damage incurred due to ferroptosis.

## INTRODUCTION

The tumor suppressor protein, p53 is a crucial factor in determining the response of cancer cells to drug treatment [1, 2]. Notably, p53 has been shown to induce different forms of cell death in cancers including ferroptosis [3–5], a form of iron-dependent cell death that results from lipid peroxidation [6]. Several reports have linked dysregulated ferroptotic death to various other diseases as well. Ferroptotic death has been implicated in multiple neurodegenerative disorders such as Huntington’s, Alzheimer’s, Parkinson’s and ischemic stroke [7]. Excessive ferroptosis has also been shown to be a key effector of cardiomyopathy [8], renal damage and failure [9, 10] and can also potentially mediate the loss of immunity against infection [11]. Each one of the abovementioned cases has been linked to aging-related disorders.

While several reports have demonstrated the ability of p53 to modulate the ferroptotic sensitivity of cancer cells, the directionality of this regulation is complex and context-specific, which is not unlike the other known stress-responses of p53 [5]. Therefore, as most of the differential responses of p53 to other stresses depend on its activation of appropriate target genes, we have examined the ability of p53 target genes to regulate ferroptosis. Since p53 can also promote premature aging [12, 13], neurodegenerative disorders [14–16] and developmental syndromes [17, 18] through its target genes, such a study would also give further insight into understanding the regulation of ferroptosis in these contexts. In line with this goal, we recently discovered that two key proteins of the p53 network, MDM2 and MDMX (the negative regulators of p53) are capable of promoting ferroptosis both in human cancer cells and in the context of neurodegeneration [19]. In the current study, we examine another well-validated target of the p53 network, p21, which is a cyclin dependent kinase that often mediates p53-induced cell cycle arrest [20].

As a consequence of the ability of p21 to induce cell cycle arrest, p21 may mediate cellular senescence, although whether p21 is a major regulator of this process is somewhat unclear [21, 22]. While stress-induced senescence is beneficial by blocking tumorigenesis due to unchecked proliferation of damaged cells, as well as by aiding tissue repair, it can also lead to undesirable effects on longevity due to prolonged accretion of senescent cells that are associated with tissue damage and aging [23].

Relatedly, in the context of some stressors, the loss of p21 has been shown to limit tissue damage and promote tissue regeneration [24] without necessarily leading to tumorigenesis [25]. On the other hand, while the function of p21 is mostly tumor suppressive, there are reports that suggest that when activated in a p53-independent manner, p21 can turn tumorigenic by protecting damaged cells from death [26]. In support of the tumorigenic potential of p21, a recent report demonstrated that the type of activation of p21 in response to chemotherapy dictates its behavior as a tumor suppressor by promoting senescence or as a tumor-driver by causing enhanced survival of so treated cancer cells [27]. In light of these conflicting roles in cancer, it is possible that the type of damage incurred would also dictate whether p21 could limit physiological tissue damage. In support of this prospect, p21 could either delay aging or promote tissue damage based on the type of tissue involved in a model of progeria [28]. Thus, examining the relationship of p21 and ferroptosis is important in the context of cancer as well as aging phenotypes.

Based on prior reports, p21 does have some potential links to ferroptosis. p21 can mediate the p53-ROS signaling pathway by helping sustain higher levels of ROS to effect senescence in some cancer cells [29]. High levels of heme-oxygenase-1 have been known to confer a resistance to apoptosis by altering cellular growth possibly due to upregulation of p21 levels [30]. It has also been shown that heme-oxygenase can enhance ferroptotic death [31, 32] but the possibility that p21 could also modulate this type of death is yet to be explored. Of direct relevance to ferroptosis, p21 has been shown to mediate the resistance of liver cells to treatment with sorafenib [33], a chemotherapeutic kinase inhibitor that has been shown to induce ferroptotic death [34]. In fact, sorafenib treatment triggers an induction of p21 and a knock-down of p21 can increase cellular killing by sorafenib [33]. Since at least a part of the death due to sorafenib can be attributed to ferroptosis, this strongly suggests a role for p21 in regulating ferroptosis. A more recent report effectively showed that p53 poses an impediment to the kinetics of ferroptosis in some human cancer cells via the p21-dependent maintenance of the intracellular glutathione pool [35].

In this study, we suggest another potential mechanism for p21 to promote tumorigenesis by serving as a barrier to ferroptosis, even in the absence of p53. In agreement with previous reports regarding the ability of p21 to enhance tumorigenesis [36], our study also shows that ferroptosis induction leads to a p53-independent regulation of *p21*. Given the prominent roles of ferroptosis in promoting organ damage, our study also supports the possibility that in damages incurred through ferroptosis, p21 could actually aid longevity instead of being a barrier to the organismal lifespan.

It is well known that the major roles of p21 in growth inhibition are mediated by its two main interactions with CDKs and the proliferating cell nuclear antigen (PCNA) [36]. The inhibitory effect of p21 on CDKs mediates its effect on the different cell cycle stages, whereas its abrogation of the role of PCNA mediates its ability to block damaged-DNA replication [37]. Since both CDKs and PCNA have roles that extend beyond just growth inhibition, p21 is able to control other processes as well. For example, p21 mediates a significant portion of the ability of p53 to repress transcription [38–40]. Further, previous reports suggest that the oncogenic role of p21 in preventing death of cancer cells is through its interaction with the CDKs [36]. Since our results reveal a potential for cyclin-dependent kinases (CDKs) to be involved in ferroptosis, they identify a new pathway involved in regulating ferroptosis.

## RESULTS

### The directionality of regulation of ferroptosis by p53 is highly context specific

**Figure 1:**
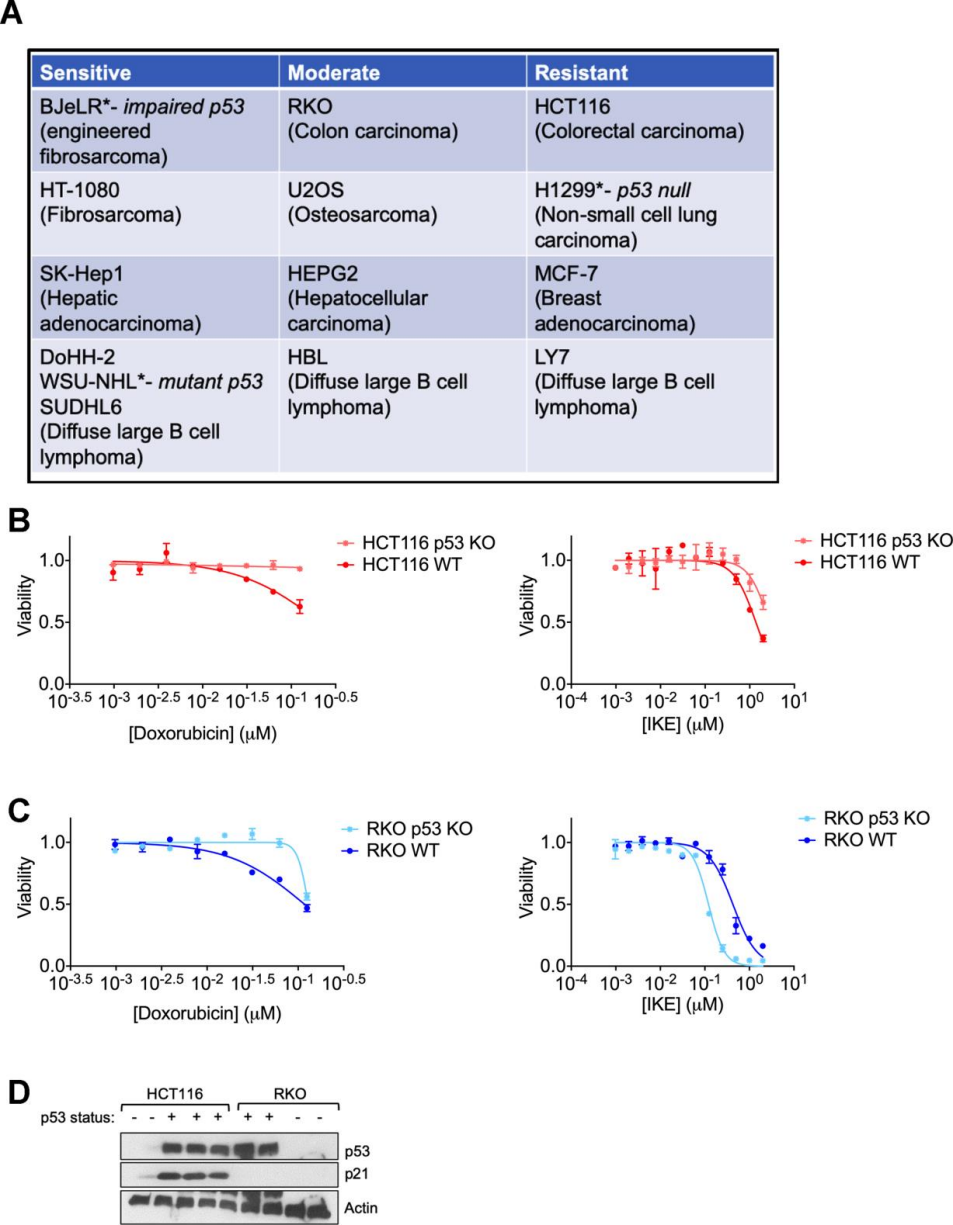
**Regulation of ferroptosis by p53 is highly context specific.** (**A**) The indicated cell lines were categorized based on the relative amount of cell death observed in response to 24 hours treatment with erastin in a 6-well format. After 24 hours of treatment, the sensitive cell lines had an EC50 of less than 2 μM of erastin, while the moderately sensitive cell lines had an EC50 that was greater than 2 μM, but lesser than 10 μM of erastin. In the resistant cell lines, erastin did not achieve 50% killing at this time point. (**B**, **C**) Viability of isogenic cell lines with wild-type (WT) p53 or no p53 (KO) in (**B**) HCT-116 and (**C**) RKO when treated with indicated doses of either doxorubicin (left panel) or IKE (right panel) for 24 hours. (**D**) Immunoblot showing p53 and p21 protein levels in HCT-116 and RKO cells. Multiple replicates of the wild-type and p53 KO cell lines cultured in separate dishes were used. Actin was used as a loading control. The data in (**B**, **C**) represent the mean ± SE for two of four independent experiments. The viability data have been normalized to that of the DMSO control.

We analyzed the response of several human cancer cell lines to the ferroptosis inducer erastin that belongs to the class I ferroptosis inducers (FINs) [41] and categorized them based on the degree of response (Figure 1A). In line with previous reports, even cell lines having the same tissue of origin varied in their response to ferroptosis [42]. The main aim of the current study was to identify if p53 or its targets could be responsible for the dichotomy between at least some of the resistant and sensitive cells. Although we had previously surmised that p53 status was not always predictive of the ferroptosis sensitivity of a given cancer cell line [19], we wanted to determine if the loss of p53 in a given cancer type would then alter its sensitivity to ferroptosis.

We chose two colon cancer cell lines with varying ferroptosis sensitivities- RKO and HCT-116 (Figure 1A) for which isogenic derivatives with respect to their p53 status were already available. These isogenic cell lines were created by the deletion of a functional domain of *p53* [43]. In both cell lines, the loss of p53 made them less sensitive to the chemotherapeutic doxorubicin (Left panels of Figure 1B, 1C), which is thought to elicit at least part of its effects on cancer cell survival through the activation of p53 [44]. On the other hand, the loss of p53 only slightly decreased the ferroptosis sensitivity of HCT-116 cells, while the RKO cells actually became more sensitive upon the loss of p53 (Right panels of Figure 1B, 1C). These results highlight the complexity in defining a set direction of regulation of ferroptosis by p53. Our findings are in line with the current literature in the field showing that p53 can either promote or block ferroptosis [5].

### p21 is differentially regulated between cells that are sensitive and resistant in response to ferroptosis

We reasoned that the nuanced roles of p53 in ferroptosis might be indirect and perhaps based on one or more p53 targets being activated in response to ferroptosis induction. To this end, we sought to examine the protein levels of p21, as it is one of the key downstream targets of p53. In fact, one key difference between the HCT-116 and RKO cells used above was their relative p21 protein abundance (Figure 1D).

**Figure 2:**
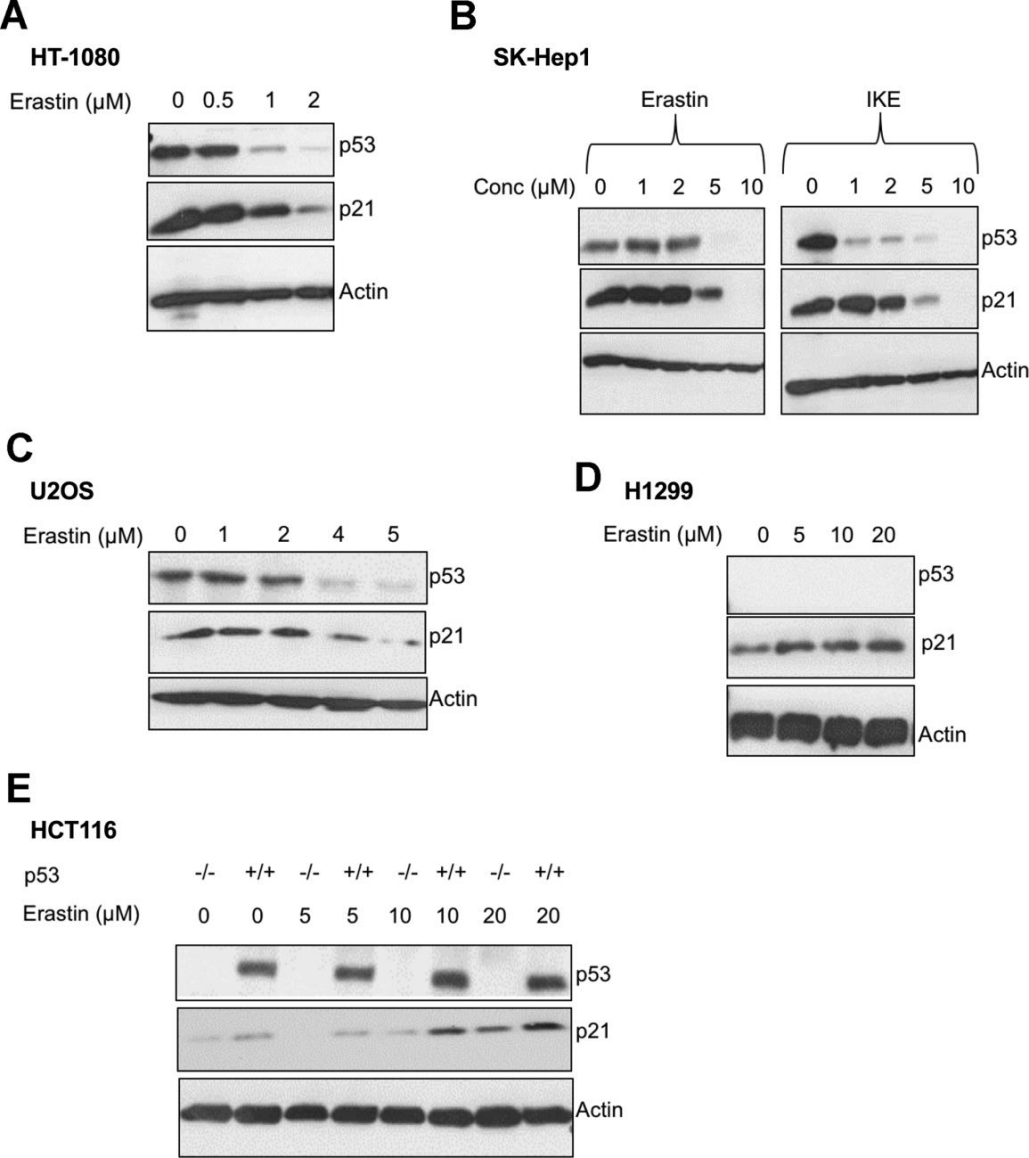
**p21 protein is differentially regulated between cells that are sensitive and resistant in response to ferroptosis.** (**A**–**E**) Impact of treatment with erastin/IKE on the protein levels of p21 and p53. (**A**) HT-1080 cells, (**B**) SK-HEP1 cells and (**C**) U2OS cells were treated for 16 hours whereas (**D**) H1299 cells and (**E**) HCT116 cells (+/+ and -/- isogenic lines with respect to p53 status) were treated for 48 hours.

We found that upon the induction of ferroptosis using two class 1 FINs (erastin or IKE), three different ferroptosis-sensitive cell lines (HT-1080, SK-HEP1 and U2OS) showed decreased levels of p21 protein (as well as p53) as a function of erastin concentration (Figure 2A–2C). On the other hand, there was an increase in p21 protein levels in two ferroptosis-resistant cell lines (HCT-116, H1299) (Figure 2D, 2E). This increase in the levels of p21 was p53-independent since it was observed even in the p53-null H1299 cell line and in HCT-116 cells that were engineered to lose p53 (p53 KO HCT116).

We also evaluated the effect of FINs on the p21 protein levels of p53 KO derivatives of the sensitive cells, HT-1080 and SK-HEP1. As we had reported previously, the loss of p53 impairs the ferroptosis-response of these cells to some extent [19]. While the HT-1080 p53 KO cells still fall within the ferroptosis-sensitive category defined in Figure 1A, the increase in ferroptosis-resistance caused by the loss of p53, places SK-HEP1 p53 KO cells on the upper edge of the moderate class. Accordingly, p21 levels were decreased in the ferroptosis-sensitive HT-1080 p53 KO upon treatment with FINs, while they were enhanced in the SK-HEP1 p53 KO cells, which moderately resist ferroptosis (Supplementary Figure 1). These results further support that the resistance to ferroptosis and the ability of the cell line to promote FIN-dependent augmentation of p21 protein levels are linked independent of the p53 status.

**Figure 3:**
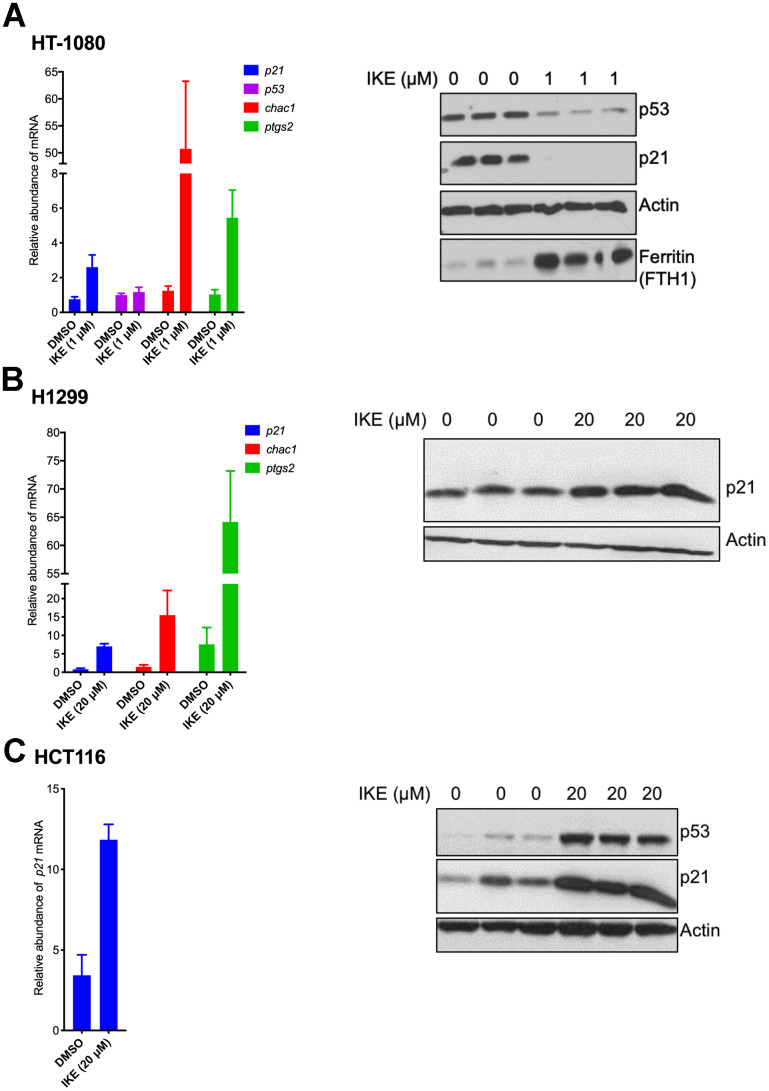
**p21 mRNA is upregulated in both ferroptosis-sensitive and ferroptosis-resistant cells after treatment with IKE.** (**A**–**C**) Left panels: Impact of IKE treatment on the mRNA levels of p21. (**A**) HT-1080 cells were treated for 16 hours while (**B**, **C**) H1299 and HCT-116 cells were treated for 48 hours. mRNA levels of *ptgs2* and *chac1* were measured in (**A**, **B**) as markers of ferroptosis. Right panels: the corresponding protein levels in the cells used in the left panels are shown. The data in left panels of (**A**–**C**) represent the mean ± SE for three biological replicates with two technical replicates each.

We then wanted to understand the nature of regulation of p21 upon ferroptosis induction. To this end we compared ferroptosis-sensitive HT-1080 (p53 wild-type) cells and ferroptosis-resistant H1299 and HCT116 cells. To our surprise, we found that in both sensitive and resistant cells, *p21* mRNA expression was upregulated at the mRNA level (Figure 3). As controls, increases in the levels of *chac1* and *ptgs2*, known to be induced during ferroptosis [6], were documented as well. Note that there was not a universal reduction in protein levels upon ferroptosis induction as evidenced by constant levels of our loading control, as well as the additional control of expected increase in levels of ferritin in ferroptosis [45]. This result indicates that the process of ferroptosis induces *p21* gene expression in a p53-independent manner and that the subsequent loss of p21 protein in the sensitive cells is most likely a consequence of a post-transcriptional event. It also suggests that this differential regulation of p21 protein may then determine the extent of death achieved.

### Altering p21 protein levels changes the sensitivity of cells to ferroptosis

The above results indicated a potential role for p21 in determining the sensitivity of cells to ferroptosis. To validate this hypothesis, we experimentally altered p21 levels and examined the changes in ferroptosis sensitivity of both resistant and sensitive cells.

**Figure 4:**
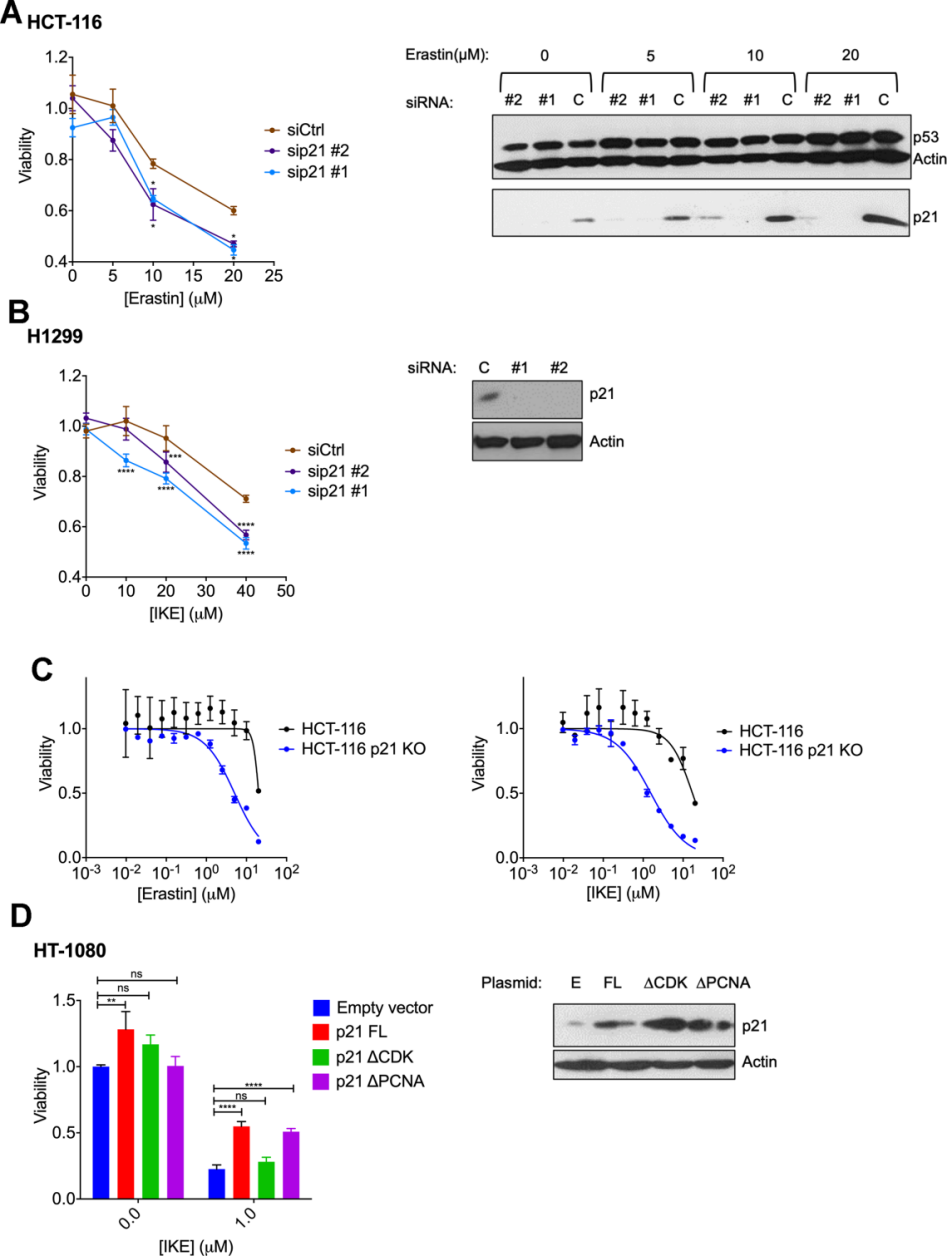
**Altering p21 protein levels changes the sensitivity of cells to ferroptosis.** (**A**, **B**) HCT116 (**A**) or H1299 (**B**) cells were transfected with two different siRNAs (#1, #2) directed against *p21* mRNA for 24 hours, and then treated with erastin or IKE as indicated for an additional 48 hours. As a control, cells were transfected with luciferase siRNA a (siCtrl/C). The right panels in A and B show the corresponding changes in p21 protein levels. (**C**) HCT-116 cells and HCT116 p21 (-/-) cells were treated with increasing doses of either erastin (left panel) or IKE (right panel) for 48 hours. (**D**) Left panel: Viability of HT-1080 cells that were transfected with the indicated plasmids expressing p21 variants or an empty vector and then treated with either DMSO or IKE for 48 hours. The panel on the right shows the corresponding immunoblot detecting p21 protein levels. The data in (**A**, **B**) represent the mean ± SE for two of three independent experiments, in (**C**) represent the mean ± SE for two out of four independent experiments, in (**D**) represent the mean ± SE for three independent experiments. The viability data have been normalized to the DMSO control in (**A**–**C**) and to their respective untreated control in (**D**).

In the resistant cell lines, HCT-116 and H1299, our goal was to determine if ferroptosis resistance can be lowered upon loss of p21. We used RNA interference against *p21* in these resistant cells and indeed observed a reduction in the resistance to ferroptosis (Figure 4A, 4B). We tested the possibility that a more complete and non-transient loss of *p21* might be required to further enhance the sensitivity of these cells, as it was reported that p21 can alter the metabolic pathways involved in ferroptosis [35]. Indeed, the HCT-116 derived *p21* -/- cell line [46], had a much-enhanced sensitivity to ferroptosis compared to its wild-type counterpart (Figure 4C).

As a reciprocal approach we increased p21 levels in the HT-1080 cell line that is ferroptosis-sensitive. Overexpression of a construct expressing wild-type p21 did suppress this form of cell death in the HT-1080 cells (Figure 4D).

Thus, the results in Figure 4A–4D demonstrate that the capacity of cells to regulate p21 when treated with FINs crucially impacts the extent of response to ferroptosis. However, it is unclear if the ferroptosis-associated reduction of p21 in sensitive cells takes place as a consequence of translational or post-translational signals. The results in Figure 4D suggests that it is unlikely for the loss of p21 protein abundance to be a result of enhanced degradation of p21, as then the ectopic p21 protein would have also faced the same fate and should then have been rendered incapable of altering the degree of ferroptotic death. In further support of this hypothesis, we observed that proteasome inhibitor MG132 is unable to block any part of the reduction in p21 expression caused by erastin (Supplementary Figure 2A left panel). Note that MG132 treatment did slightly increase the sensitivity of ferroptosis, although this was found to be p21 independent (Supplementary Figure 2A right panel). Thus, based on these results we posit that p21 is more likely to be translationally regulated in response to ferroptosis.

The experiment in Figure 4D, further allowed us to determine which interactions of p21 may aid its role in ferroptosis. For this, we used two key mutant versions of p21, which disable either its CDK binding or PCNA binding domains [47, 48] and found that they differed in their ability to suppress ferroptosis. Specifically, the CDK binding-defective version of p21 was unable to block ferroptosis, while mutating the PCNA binding region impaired p21 to a much lesser extent in that regard (although the levels of expression of this mutant were slightly lower). This result suggests the possibility that p21 alters sensitivity to ferroptosis by affecting CDK-mediated functions.

Taken together, we demonstrate that differential regulation of p21 protein can serve as a determining factor of ferroptosis sensitivity in many human cancer cells and that this regulation is independent of p53. Our results further emphasize the importance of p53-targets in the regulation of cell survival, even in the absence of p53.

## DISCUSSION

There is growing literature on the complex roles of p53 in ferroptosis [5]. Given the highly context-specific regulation of ferroptosis by p53, we focused on elucidating the ability of p53-target genes to regulate this form of cell death. In line with that, our study has revealed the ability of p21, a major downstream target of p53, to block ferroptosis in several cancer cell lines. We demonstrate that cells which effectively undergo ferroptosis reduce the expression of p21 protein. Further the sensitivity of these cells can be suppressed by re-expressing p21, suggesting that the loss of p21 is essential to allow for a complete response to ferroptosis. Conversely the resistant cells that we tested were dependent on the presence of p21 to counteract ferroptotic death. Taken together, we believe that at least in some cancer cells, the regulation of p21 protein can be the determining factor of their ferroptotic sensitivity.

Although we sought to identify p53-target genes that may help determine the directionality of the regulation of p53 in ferroptosis, this work complements our previous work [19] in identifying members of the p53 network, namely p21, MDM2 and MDMX, which can modulate ferroptosis independent of p53. It is certainly possible that these proteins perhaps coordinate with p53 to ultimately dictate the ferroptosis-sensitivity in some contexts.

Mechanistically, our results indicate that the ability of p21 to interact with CDKs is important for its role in ferroptosis. The inhibition of CDK activity by p21 can have multiple effects that impact cellular growth including altered transcription, cell cycle changes and even dedifferentiation to a certain degree [36]. Our finding that complete ablation of *p21* has a more pronounced change in ferroptotic sensitivity than a transient yet highly effective siRNA against *p21,* suggests that the mechanism of resistance likely requires prolonged presence of p21. For example, if cancer cells underwent some extent of p21-dependent dedifferentiation in order to become ferroptosis-resistant, then it is likely that these changes would need more time to get reverted. Reports showing that dedifferentiation of melanoma cells as well as further differentiation of neurons enhance ferroptosis sensitivity [49, 50] lend some support to this theory.

We speculate that cell cycle changes alone may not explain the role of p21 in ferroptosis. Relatedly, it was reported that while that p53 can prevent ferroptosis through p21, cell cycle arrest alone is insufficient to cause this suppression [35]. It is definitely possible that p21 has a myriad of effects with cell cycle changes just being a subset of them. Taken together, a future study to better understand the molecular regulation of ferroptosis by p21, should evaluate the involvement of CDKs as a key factor. It is also unclear which proteins/pathways control the regulation of p21 in response to ferroptosis, both at the transcriptional and post-transcriptional levels. Studying these could further yield more regulators of ferroptosis.

Our data also indicate that p21 can have a potential to be used as a biomarker for ferroptosis sensitivity of cancer cells. If not merely the abundance of p21 protein, the protein levels of p21 post ferroptosis induction strongly track with the sensitivity of a wide range of cancer cell lines tested. Therefore, this study identifies another important regulator of ferroptosis sensitivity in cancer.

Our data also sparks the need to further examine the role of p21 in mediating the ability of ferroptosis to cause organ damage. If p21 can indeed control the differentiation of cells through CDKs as hypothesized above, then this provides a potential mechanism for p21 to promote tissue regeneration by inhibiting ferroptosis even in physiological conditions. While this is counter-intuitive to the traditional roles of p21 in aging, it adds a new perspective to the wide variety of roles that can be played by p21 in multiple contexts.

## MATERIALS AND METHODS

### Cells

HCT116, H1299, SK-HEP1, and U2OS cells were maintained in Dulbecco’s modified Eagle’s medium supplemented with 10% heat-inactivated fetal bovine serum (Gemini Bioproducts, cat# 900-108). HT-1080 cells were maintained in Dulbecco’s modified Eagle’s medium supplemented with 10% heat-inactivated fetal bovine serum (Gemini Bioproducts, cat# 900-108), and 1% non-essential amino acids (Sigma-Aldrich, cat# M7145). RKO cells were grown in McCoy’s 5A modified medium (Gibco, cat# 16600-082) supplemented with 10% heat-inactivated fetal bovine serum (Gemini Bioproducts, cat# 900-108). The HCT116 and RKO isogenic cell lines that were created by the deletion of a functional domain of *p53* [43] were a gift from Dr. Vogelstein. The HT-1080 p53 KO, HT-1080 p21 KO and SK-HEP1 p53 KO cell lines were genetically engineered using CRISPR technology [19]. All other cell lines were obtained from ATCC.

### Drugs and chemicals

The commercially available compounds used were: erastin (Selleckchem, cat# S7242), doxorubicin (Sigma-Aldrich, cat#D1515) and MG132 (Selleckchem, cat# S2619). IKE (imidazole ketone erastin) was synthesized as in Larraufie MH et al., by Yan Zhang in Stockwell lab [51].

All the compounds were dissolved in DMSO (Sigma-Aldrich, cat# D8418).

### Quantitative reverse transcription PCR

RNA was isolated from cells using the Qiagen RNeasy minikit. cDNA was generated using the Qiagen Quantitect reverse transcription kit with 0.5 μg of input RNA as measured with a NanoDrop (Thermo Scientific). Real-time PCR was carried out on an ABI StepOne Plus machine using the power SYBR Green dye (Thermo Scientific). Transcript levels were assayed in triplicate and normalized to *L32* mRNA levels. Relative changes in cDNA levels were calculated using the comparative Ct method (ΔΔC_T_ method).

### Primer sequences

L32 F: TTCCTGGTCCACAACGTCAAG, L32 R: TGTGAGCGATCTCGGCAC; p21 F: GGCGGCAGACCAGCATGACAGATT, p21 R: GCAGGGGGCGGCCAGGGTAT; chac1 F: GAACCCTGGTTACCTGGG, chac1 R: CGCAGCAAGTATTCAGGTGT; ptgs2 F: TAAGTGCGATTGTACCCGGAC, ptgs2 R: TCTCCAAAGGAGGTTACCTGC.

The p53 primer was obtained as premixed solution from Qiagen (Quantitech primer, HS_TP53_1_SG, cat# QT00060235) and the rest were individually ordered from Invitrogen.

### Immunoblot

Cells were lysed with TEB lysis buffer (10mM Tris HCL pH 7.5-8, 137 mM sodium chloride, 10% glycerol, 1% NP-40) supplemented with 1mM magnesium chloride, 1mM calcium chloride and protease inhibitors (Roche). Protein concentrations were assayed using the Bio-Rad protein assay dye reagent and results were read using a spectrophotometer.

Protein extracts were run on in-house made Tris-Glycine SDS Polyacrylamide gels. Proteins were then electro-transferred at 360 mA for 70 min onto a nitrocellulose or PVDF membrane. Membranes were blocked with 5% milk in PBST (Phosphate-Buffered Saline with Tween) for 30 min, prior to being incubated overnight with primary antibodies (1:100-1:1000 dilution according to the specific antibody). The membranes were then washed three times with PBST and incubated with secondary antibody (1:5000 dilution) for 1 hour at room temperature. After three more washes with PBST, the membranes were imaged using ECL (Thermo Fisher, Pierce, cat# 32106 or EMD Millipore, Immobilon, cat# WBKLS0050). The primary and secondary antibodies were diluted with 1% milk in PBST.

The following primary antibodies were used: p53 (mAb 1801/mAb DO.1, in-house produced); p21 (C-19, Santa Cruz biotech, cat# sc-397); actin (Sigma-Aldrich, cat# A2066); ferritin/FTH1 (Cell Signaling Technology cat# 3998). Actin was used as loading control for all the blots.

### Transfection: RNA interference

siRNA (15 nM) was used for each well in a 6-well plate. Lipofectamine RNAiMAX (Thermo Scientific) was used as the transfection reagent for all siRNA experiments (according to the manufacturer's instructions). After 18 hours, the media was changed and cells were treated with drugs 24 hours post transfection. Cells were plated prior to transfection such that they were only 80% confluent by the end of the drug treatment period.

The following siRNAs were used: siLuciferase [52], sip21 #1 (HS_CDKN1A_6 Flexitube siRNA from Qiagen), sip21 #2 (HS_CDKN1A_7 Flexitube siRNA from Qiagen).

### Transfection: Ectopic expression of proteins

Plasmids were transfected into cells using Lipofectamine 3000 (Thermo Scientific) according to the manufacturer's instructions, with a ratio of 1 μg:1.7 μl lipofectamine reagent. After 18 hours, the media was changed and cells were treated with drugs 24 hours post transfection. The cells were plated prior to transfection such that they are only a maximum of 80% confluent by the end of the drug treatment period. The plasmids for full length and mutants of p21 were a kind gift from Dr. Vanessa Gottifredi and have been previously described [47, 48].

### Note

Cells became more resistant to ferroptosis inducers post transfection. In order to obtain cell death post transfection, three key factors need to be controlled: cell density must be lower than normal, lipofectamine reagent needs to be washed off as soon as possible, and a three to four-fold higher dose of FINs must be used to induce ferroptosis.

### Cell viability assay

For the dose response curves, 1800 cells were plated in 36 μl per well of a 384 well plate on day one. Drugs were dissolved in DMSO and a 12 point, two-fold series was prepared. The drugs were then dissolved 1:33 in media and 4 μl was added to each well of the plates on day two. After 24-48 hours of drug treatment (based on the cell line), the viability of cells was measured using a 1:1 dilution of the CellTiter-Glo Luminescent reagent (Promega, cat# G7573) with media, which was read on a Victor 5 plate reader after 10 minutes shaking at room temperature. The intensity of luminescence was normalized to that of the DMSO control. Experiments were performed twice in duplicates each time.

For viability assays when the experiment was performed in 6-well plates, cells were harvested using trypsin (0.5 ml per well) and the media was saved from each well. The trypsinized cells were resuspended with the saved media and 2-3 aliquots (0.05 ml each) sampling different regions of this suspension were transferred into a 96-well plate to serve as technical replicates for the measurement. CellTiter-Glo Luminescent Viability assay was used to measure the viability of these aliquots. The rest of the cultures were used to extract protein to be analyzed using western blots.

### Statistical analysis

Prism (version 8, GraphPad) was used to make all the graphs in the paper and for performing all the statistical analysis shown. The GraphPad style (0.1234(ns), <0.0332(*), < 0.0021(**), <0.0002(***), <0.0001(****),) was used to represent the p values. The p values were calculated by ANOVA and appropriate multiple testing correction was done where required.

## Supplementary Material

Supplementary Figures
