# Supplementary material for: p21 can be a barrier to ferroptosis independent of p53
Source: Aging (Albany NY). 2020 Sep 24;12(18):17800–14. doi: 10.18632/aging.103961 (PMC7585094; doi:10.18632/aging.103961)
Supplement: Supplementary Figures [file aging-12-103961-s001..pdf]

SUPPLEMENTARY FIGURES

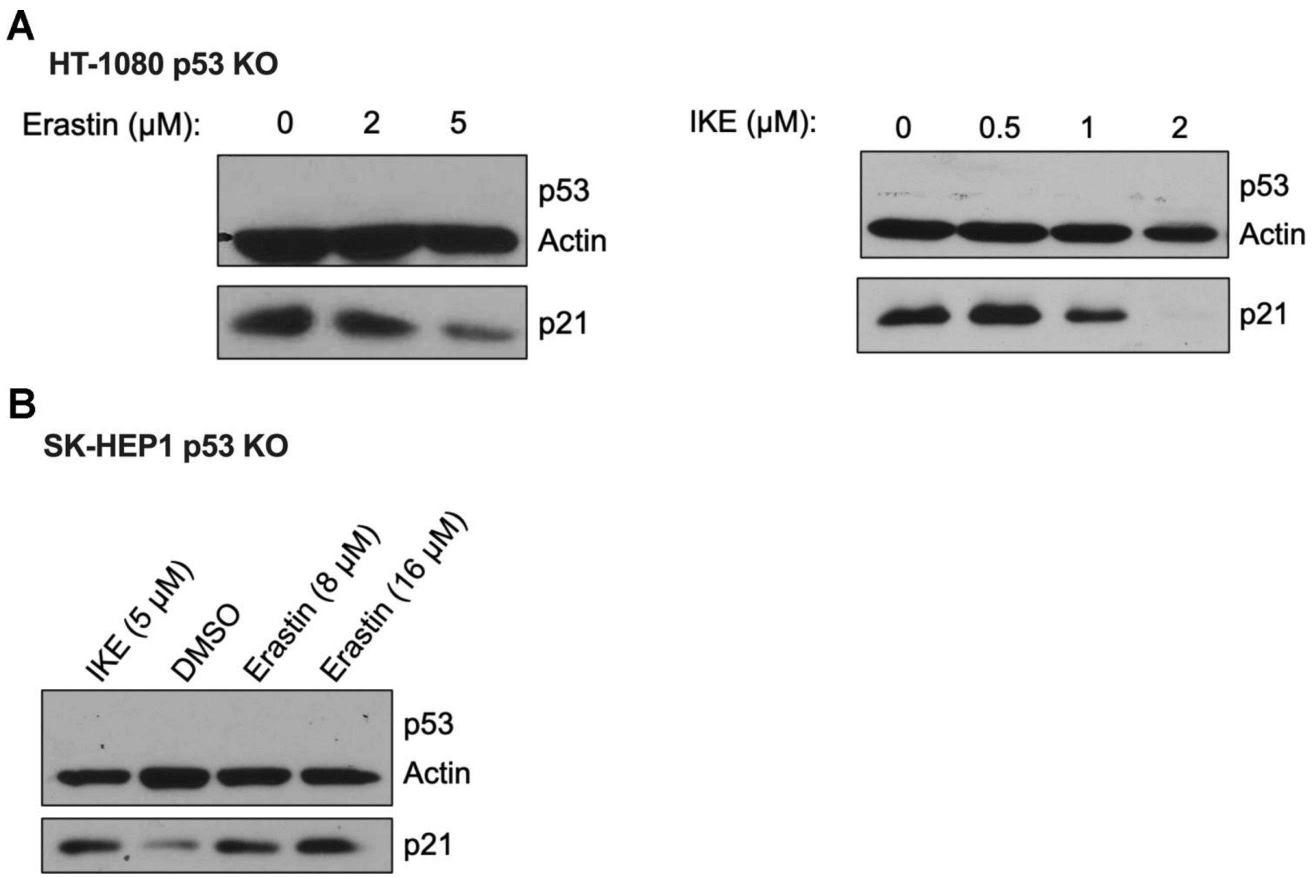

Supplementary Figure 1. Ferroptosis-driven differential regulation of p21 protein is independent of p53. (**A**, **B**) Impact of treatment with erastin/IKE on the protein levels of p21in (**A**) HT-1080 p53 KO cells and (**B**) SK-HEP1 p53 KO cells. Cells in (**A**) were treated with erastin/IKE for 16 hours and in (**B**) for 18 hrs.

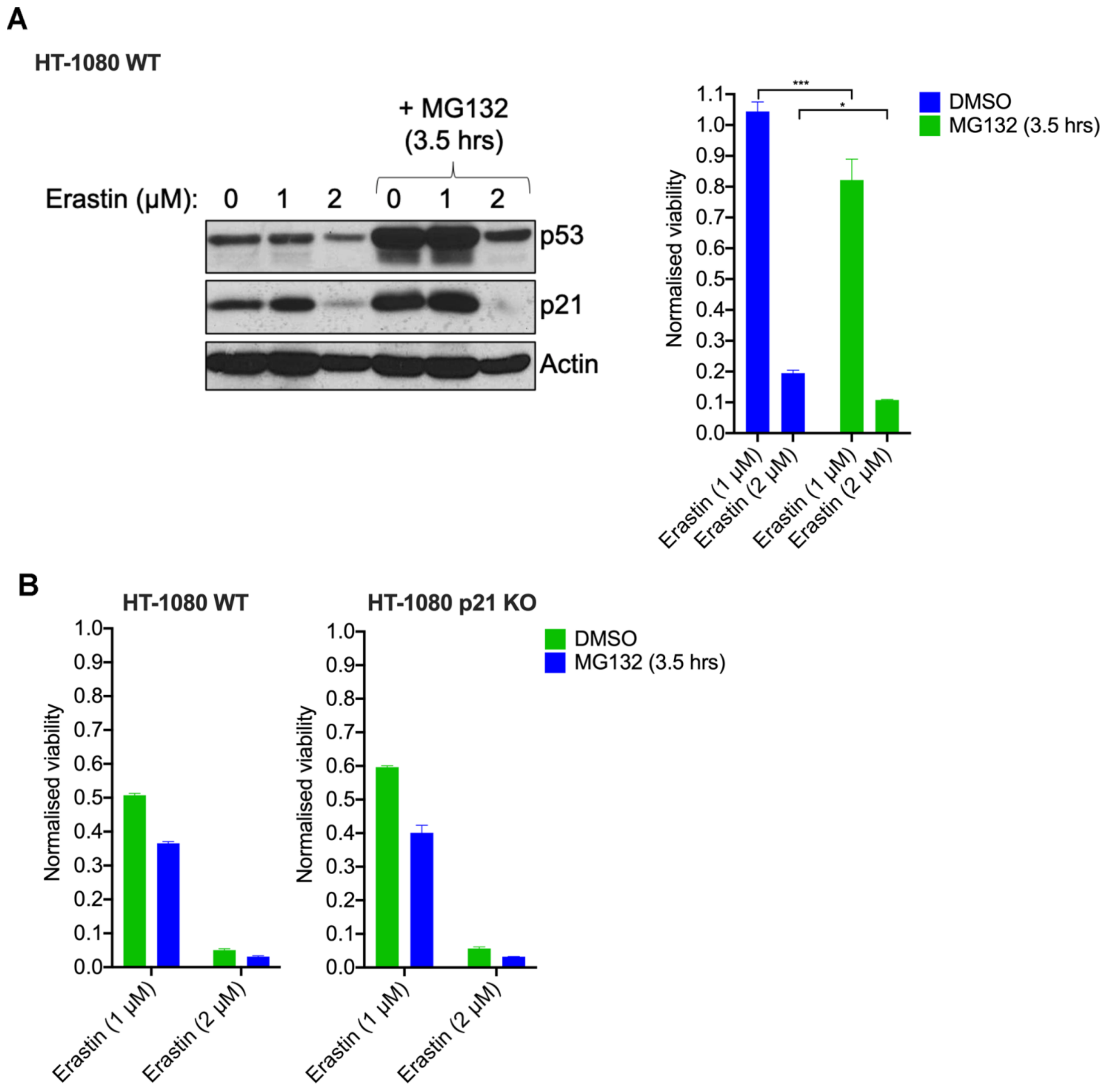

Supplementary Figure 2. Suppression of the proteasome does not revert the reduction in p21 protein levels due to ferroptosis. (**A**) Left panel- effect of addition of MG132 on protein levels of p21 and p53 in HT-1080 wild-type cells treated with erastin. Right panel- viability of HT-1080 wild-type cells when treated with MG132 in conjugation with erastin. (**B**) Comparison of responses of HT-1080 wild-type and p21 KO derivatives to combination treatment of erastin and MG132. Cells were treated with erastin for 16 hrs and MG132 (20μM) was added after 12.5 hours post erastin treatment. The data in right panel of (**A**) represent the mean ± SE for three biological replicates of one representative of three independent experiments, in (**B**) represent the mean ± SD for one out of two independent experiments. The viability data have been normalized to the respective controls not treated with ferroptosis.
